# Association of early-life undernutrition and risk of dyslipidemia in adulthood: a population-based cohort study

**DOI:** 10.1186/s12889-021-12211-8

**Published:** 2021-11-20

**Authors:** Minmin Wang, Mengfei Liu, Chuanhai Guo, Fenglei Li, Zhen Liu, Yaqi Pan, Fangfang Liu, Ying Liu, Huanyu Bao, Zhe Hu, Hong Cai, Zhonghu He, Yang Ke

**Affiliations:** 1 Key Laboratory of Carcinogenesis and Translational Research (Ministry of Education/Beijing), Laboratory of Genetics, Peking University Cancer Hospital & Institute, #52 Fucheng Rd, Haidian District, 100142 Beijing, China; 2 Hua County People’s Hospital, Anyang, Henan Province China

**Keywords:** Undernutrition, Great Chinese Famine, Dyslipidemia, Confounding effect

## Abstract

**Background:**

The association of early-life undernutrition and dyslipidemia found in previous studies may be confounded by the uncontrolled age difference between exposed and unexposed participants. The study aimed to investigate the association of early-life undernutrition and the risk of dyslipidemia in adulthood with good control of the age variable.

**Methods:**

We took the Great Chinese Famine (1959–1961) as a natural experiment of severe undernutrition. This study was based on the baseline investigation of a population-based cohort in rural China. Undernutrition in early life was defined as being exposed to famine at younger than 3 years of age. Three approaches including Adjustment, Restriction, and Matching were applied to control the confounding effect of age. Logistic regression models were applied to evaluate the association between early-life famine and the presence of dyslipidemia. Stratified analysis by gender was also performed, and potential effect modification was tested by adding the interaction term of the famine exposure variable and gender into the model.

**Results:**

Undernutrition in early life was associated with increased risk of borderline high and above (BHA) levels of total cholesterol (TC, OR_Adjustment_ = 1.61; OR_Restriction_ = 1.56; OR_Matching_ = 1.87), triglycerides (TG, OR_Adjustment_ = 1.33; OR_Restriction_ = 1.30; OR_Matching_ = 1.34), low-density lipoprotein cholesterol (LDL-C, OR_Adjustment_ = 1.75; OR_Restriction_ = 1.53; OR_Matching_ = 1.77) and dyslipidemia (OR_Adjustment_ = 1.52; OR_Restriction_ = 1.45; OR_Matching_ = 1.60), as well as high levels of TC, TG, LDL-C and dyslipidemia. An inverse association of undernutrition and risk of low high-density lipoprotein cholesterol (HDL-C) was found. Female participants with undernutrition experience had an increased risk of BHA TG and LDL-C (TG: OR_Adjustment, female_ = 1.45; OR_Restriction, female_ = 1.39; OR_Matching, female_ = 1.51; LDL-C: OR_Adjustment, female_ = 2.11; OR_Restriction, female_ = 1.80; OR_Matching, female_ = 2.15), but this association was not found in males.

**Conclusion:**

Early-life undernutrition increased the risk of TC, TG, LDL-C, and dyslipidemia. Gender would significantly modify this effect for TG and LDL-C. These results emphasize the importance of nutritional conditions in the early stages of life to long-term health consequences.

**Supplementary Information:**

The online version contains supplementary material available at 10.1186/s12889-021-12211-8.

## Background

Mortality associated with cardiovascular disease (CVD) has risen in China over the last decade, and CVD accounted for over 40% of deaths from all causes in 2014 [1]. Lipid abnormalities are a principal contributor to CVD, and the prevalence of dyslipidemia is over 30% in rural residents in China [2–5]. Dyslipidemia is a long-term result of the combined effects of genetic factors and environmental exposure (such as unhealthy diet and lifestyle). Exposure to undernutrition in early life will likely have a long-term influence on lipid metabolism mode [6–8].

China experienced a serious famine in 1959–1961, which caused millions of excess deaths with an annual mortality of over 3.0% during the years of famine [9–11]. The Great Chinese Famine was characterized by long duration, a large affected geographic area, and the serious consequences it engendered. In the current era, most middle-aged Chinese were exposed to this famine at some point in their early life.

There have been several studies that have taken the 1959–61 Chinese famine as a natural experiment to investigate the association of severe undernutrition and the onset of dyslipidemia in adulthood. As it is difficult to establish an “unexposed control” in parallel with the famine-exposed cohort, previous studies typically applied a “birth cohort” design, where the control cohorts are selected from individuals of post-famine birth who are at least 3–5 years younger than the exposed subjects. The association of undernutrition and the risk of dyslipidemia is then evaluated by comparing the prevalence of dyslipidemia in these two cohorts. However, under this study design, it is of concern that the estimated undernutrition effect might be confounded by age, as age is strongly associated with undernutrition exposure as well as with the risk of dyslipidemia [4]. Given the fact there is almost no overlap in the age range in the two birth cohorts, using multivariable models in traditional statistical methods may not be sufficient to diminish the confounding effect of age. One meta-analysis [12] suggested that most of the associations observed in undernutrition and metabolic syndrome would turn null if age was additionally matched when selecting the control cohorts. This revealed that uncontrolled age differences might explain some aspects of the effect attributed to undernutrition in previous studies. As a result, age differences in undernutrition exposed and unexposed cohorts should be controlled as the priority in studies investigating the association between early-life undernutrition and long-term health risk.

In this study, we applied three approaches for analysis, namely Adjustment, Restriction, and Matching to control for the confounding effect of age, and then investigated the association of early-life undernutrition exposure and risk of dyslipidemia in adulthood based on the baseline investigation of a population-based cohort in rural China.

## Methods

### Population

This study was conducted in Hua County of Henan Province, China, which covered an area of 700 mile^2^. This area is predominantly rural and 90% of the total population is involved in agriculture. Henan province suffered severe food shortages during the Great Famine. The mortality in 1960 reached 39.6‰, which was two-fold higher than the average level in 1956–58 [9, 13]. The population sizes of individuals of 51–53 years (born in 1959–1961) decreased about 50% compared with the population of 54–56 years (born in 1956–1958) according to the population statistics in 2012 [14].

The study enrolled participants aged 45–69 years from 668 randomly selected villages in Hua County based on the baseline investigation of Endoscopic Screening for Esophageal Cancer in China (*ESECC*) randomized controlled trial (ClinicalTrials.gov identifier: NCT01688908). The design and preliminary results of the “*ESECC*” trial can be found elsewhere [15]. Briefly, 668 out of 846 villages in rural Hua County with population size ranging from 500 to 3000 were randomly selected and permanent residents aged 45–69 in each of the target villages were invited to participate in the trial on a voluntary basis. A total of 35,772 permanent residents were recruited during the period December 2011–September 2016.

All participants in this study were measured for height and weight at enrollment. Participants’ seated blood pressure was measured after 5 min of rest with a mercury sphygmomanometer. A computer-aided one-on-one questionnaire was completed by well-trained interviewers to collect information on personal characteristics, socioeconomic status, lifestyle factors and health status. A fasting blood sample of ~ 5 mL was then collected from each participant in a heparin sodium anticoagulant tube. These tubes were then centrifuged at 1000 rpm for 5 min and the supernatants were sent within 4 h for measurement of total cholesterol (TC), low-density lipoprotein cholesterol (LDL-C), high-density lipoprotein cholesterol (HDL-C) and triglycerides (TG) at the clinical laboratory of Hua County People’s Hospital. Lipid measurements were conducted using a HITACHI7600 automatic biochemistry analyzer (Hitachi High Technologies Co., Tokyo, Japan) with commercially available reagents (Autobio Diagnostics Co., Ltd., Beijing, China). All experiments were performed in accordance with relevant guidelines and regulations. Detailed information on the measurement of lipids can be found elsewhere [4].

### Study design

Undernutrition exposure was defined based on participants’ date of birth. Individuals who were under the age of 3 during the 1959–1961 Great Chinese Famine (i.e., born between 1 January 1956 and 31 December 1961) were defined as having early-life undernutrition. The non-exposed cohort was defined as participants born between 1 January 1963 and 31 December 1968 with one year after the famine as a washout period to minimize misclassification.

Eligibility criteria for the current study included: 1) “*ESECC*” cohort member; 2) member of the defined undernutrition exposed or non-exposed cohorts; 3) signed informed consent for participation; 4) questionnaire completed; 5) blood samples provided, with valid test results for blood lipids.

**Fig. 1:**
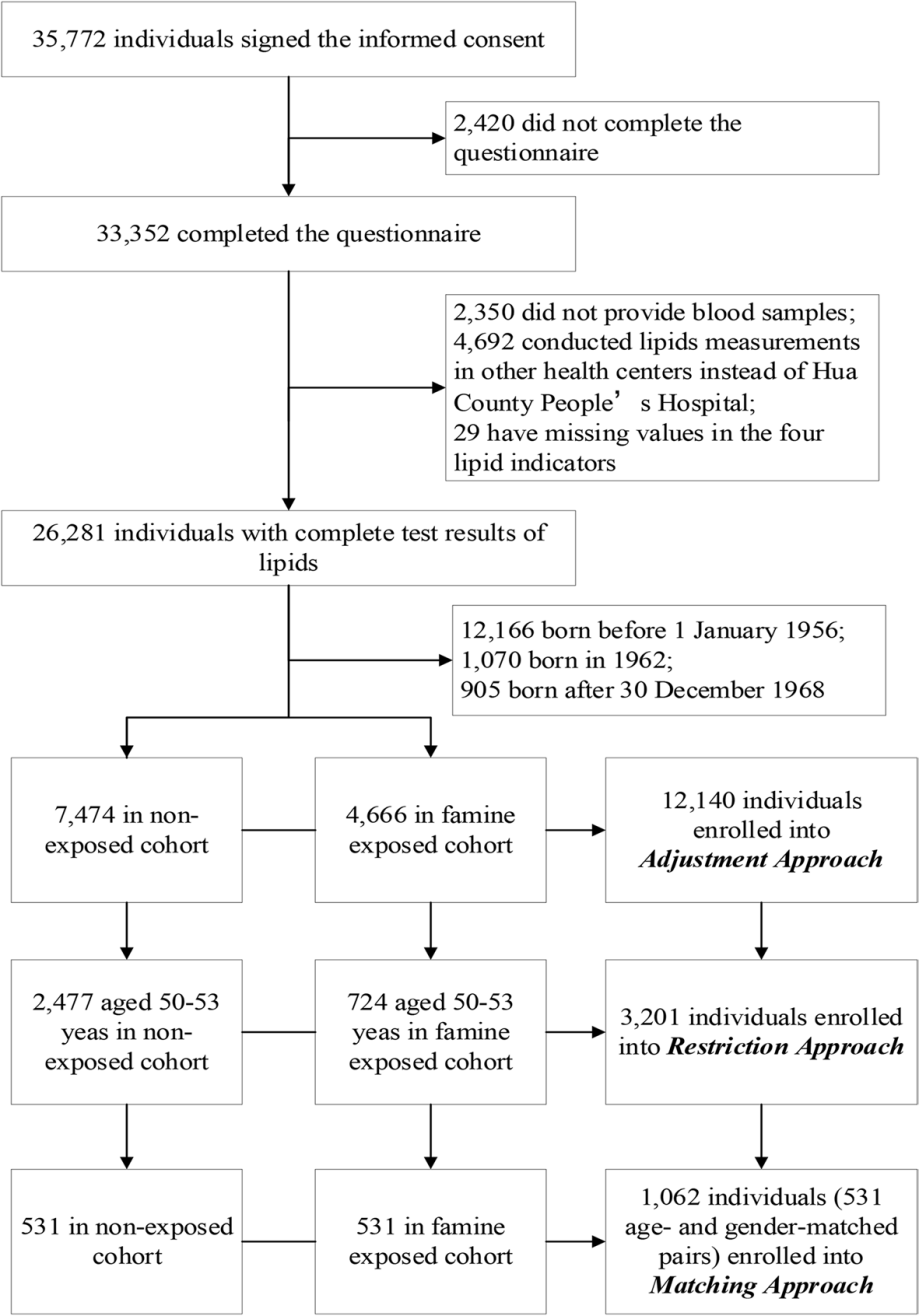
Flowchart for participants enrolled in three analytic approaches

Distinct from other cross-sectional studies, the baseline investigation of the “*ESECC*” continued from 2011 to 2016. Due to the five-year enrollment process, participants in both the undernutrition-exposed and non-exposed cohorts had a relatively wide age range at enrollment where individuals were at 50–60 years and 43–53 years in the exposed and non-exposed cohort, respectively. The overlap in age at enrollment provided an opportunity to control the confounding effect of age through three analytical approaches. First, under the Adjustment Approach, all participants who fulfilled the eligibility criteria were included, and a multivariable model was adopted to adjust the age variable as in previous studies [16, 17]. Secondly, the Restriction Approach included only participants aged 50–53 years (the overlapped age range for undernutrition-exposed cohort and non-exposed cohort) in order to decrease the age difference between cohorts before statistical adjustment. The multivariable model was also applied to further diminish the confounding effect of age. Lastly, under the Matching Approach, individual-level matching was applied to completely rule out the confounding effect of the age variable where each subject in the undernutrition-exposed cohort was matched to a subject from the non-exposed cohort by age and gender. These three approaches showed an increasingly strong capacity to control age differences between cohorts despite decreased sample size (Fig. 1).

### Definition of dyslipidemia

**Fig. 2:**
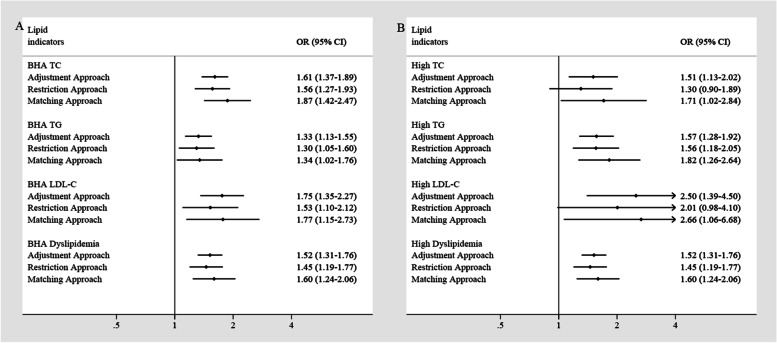
Estimates of association of early-life undernutrition and risk of dyslipidemia in three analytic approaches*. **A** Association of early-life undernutrition and risk of borderline high and above (BHA) status of dyslipidemia in adulthood. **B** Association of early-life undernutrition and risk of high status of dyslipidemia in adulthood. *ORs were adjusted for age, occupation, BMI, blood pressure, cigarette smoking, alcohol drinking, fried food intake, salty food intake, spicy food intake, heartburn and regurgitation, and self-reported history of diabetes

According to the guidelines for the prevention and treatment of dyslipidemia in Chinese adults (2016 version), borderline high TC was defined as ≥200 mg/dL (5.2 mmol/L) and <240 mg/dL (6.2 mmol/L); high TC as ≥240 mg/dL (6.2 mmol/L); borderline high TG as ≥150 mg/dL (1.7 mmol/L) and <200 mg/dL (2.3 mmol/L); high TG as ≥200 mg/dL (2.3 mmol/L); borderline high LDL-C as ≥130 mg/dL (3.4 mmol/L) and <160 mg/dL (4.1 mmol/L); high LDL-C as ≥160 mg/dL (4.1 mmol/L); reduced HDL-C as <40 mg/dL (1.0 mmol/L). In view of discrepancies in age distribution [4] and in the identified association of HDL-C and other lipid indicators (Fig. 2, Supplementary Fig. 1), dyslipidemia was defined based only on TC, TG and LDL-C. Two sets of definitions for dyslipidemia were used in this study, namely borderline high and above (BHA) dyslipidemia, and high dyslipidemia. BHA dyslipidemia was defined as the presence of a high level or borderline high level of any factor including TC, TG and LDL-C, and high dyslipidemia was defined as the presence of a high level of any one of these three lipid molecules in a given study subject.

### Covariates

Risk factors for dyslipidemia identified in the previous study were collected as covariates [4], including age, occupation (physical or nonphysical worker), body mass index (BMI: normal, overweight, or obesity), blood pressure (normal, or hypertension), cigarette smoking (no smoking, moderate-smoker, and heavy-smoker), alcohol drinking (no drinking, moderate-drinker, and heavy-drinker), fried food intake (seldom intake, occasional or often intake), salty food intake (seldom intake, occasional or often intake), spicy food intake (seldom intake, occasional or often intake), heartburn and regurgitation, and self-reported history of diabetes. Detailed information on the definitions and coding forms of the covariates were listed in Supplementary Table 1.

### Statistical analysis

The Chi-square test and Student t-test were used to compare demographic characteristics and behavioral factors in exposed and non-exposed cohorts. Logistic regression models were applied to evaluate the association of early-life undernutrition experience and the presence of dyslipidemia. In multivariable models, covariates of risk factors for dyslipidemia were adjusted. The identified associations were further validated in subgroup analysis stratified by BMI groups given the strong association of BMI and dyslipidemia. Considering the gender heterogeneity in dyslipidemia prevalence, trends with age and associated factors [4], we estimated the association in stratification of gender. Potential interaction between undernutrition exposure and gender was tested by adding an interaction term of the undernutrition variable and gender into the model.

Statistical analysis was performed using STATA (Version 13.1; Stata Corp LLC, TX, USA). All tests were two-sided and *P* values < 0.05 were considered statistically significant.

## Results

From the baseline investigation of the *ESECC* trial, 7474 participants in the exposed cohort and 4666 in the non-exposed cohort were eligible for the current study. All 12,140 eligible individuals were enrolled into the Adjustment Approach, and from this group, 3201 individuals aged 50–53 years (2477 from the undernutrition exposed cohort and 724 from the non-exposed cohort) were enrolled into the Restriction Approach; and 531 age- and gender-matched pairs (1062 individuals) were included in the Matching Approach (Fig. 1).

Key demographic characteristics and behavior variables were compared in the exposed and non-exposed cohorts using three separate analytic approaches (Table 1, Supplementary Table 2). In the Adjustment Approach, the mean age of enrolled participants was 48.78 years for the non-exposed cohort and 55.86 years for the exposed cohort. In the Restriction Approach, the mean age was 50.94 and 52.16 years for non-exposed and exposed subjects. In individual-level matching, the mean age was 51.85 years for both cohorts. The exposed and non-exposed cohorts showed significant differences in BMI, prevalence of hypertension, and intake of spicy food. These two groups of participants did not show marked differences in other behavioral or lifestyle variables. Regarding the distribution of lipid indicators, undernutrition exposed subjects had a significantly higher level of TC and HDL-C compared with non-exposed participants across these three analytic approaches. The undernutrition exposure was also associated with a significantly higher level of TG under the Restriction and Matching Approach. For LDL-C, the Adjustment Approach demonstrated a significant difference in these two groups but not in the other two approaches.

**Table 1:** Distribution of age, gender and four lipid indicators in three analytic approaches

	Adjustment Approach^a^			Restriction Approach^b^			Matching Approach^c^		
	Non-exposed cohort (*N* = 7474)	Undernutrition exposed cohort (*N* = 4666)	*P* value^d^	Non-exposed cohort (*N* = 2477)	Undernutrition exposed cohort (*N* = 724)	*P* value^d^	Non-exposed cohort (N = 531)	Undernutrition exposed cohort (*N* = 531)	*P* value^d^
Age									
Mean (SD)	48.78 (2.12)	55.86 (2.23)	< 0.001	50.94 (0.92)	52.16 (0.95)	< 0.001	51.85 (0.94)	51.85 (0.94)	–
Gender									
Male	3422 (45.79)	2213 (47.43)	0.077	1149 (46.39)	320 (44.20)	0.299	241 (45.39)	241 (45.39)	–
Female	4052 (54.21)	2453 (52.57)		1328 (53.61)	404 (55.80)		290 (54.61)	290 (54.61)	
TC level (mmol/L)									
Mean (SD)	4.69 (0.88)	4.86 (0.91)	< 0.001	4.68 (0.88)	4.91 (0.91)	< 0.001	4.61 (0.85)	4.92 (0.93)	< 0.001
TC categories									
Ideal	5545 (74.19)	3103 (66.50)	< 0.001	1842 (73.64)	475 (65.61)	< 0.001	410 (77.21)	347 (65.35)	< 0.001
Borderline High	1533 (20.51)	1200 (25.72)		518 (20.91)	190 (26.24)		94 (17.70)	139 (26.18)	
High	396 (5.30)	363 (7.78)		135 (5.45)	59 (8.15)		27 (5.08)	45 (8.47)	
TG level (mmol/L)									
Mean (SD)	1.62 (1.50)	1.57 (1.16)	0.099	1.56 (1.20)	1.67 (1.42)	0.039	1.50 (0.85)	1.72 (1.56)	0.004
TG categories									
Ideal	5171 (69.16)	3218 (68.97)	0.309	1718 (69.36)	481 (66.44)	0.307	365 (68.74)	346 (65.16)	0.017
Borderline High	1168 (15.63)	771 (16.52)		410 (16.55)	128 (17.68)		106 (19.96)	93 (17.51)	
High	1135 (15.19)	677 (14.51)		349 (14.09)	115 (15.88)		60 (11.30)	92 (17.33)	
LDL-C level (mmol/L)									
Mean (SD)	2.45 (0.62)	2.54 (0.66)	< 0.001	2.48 (0.59)	2.53 (0.71)	0.107	2.49 (0.60)	2.52 (0.74)	0.402
LDL-C categories									
Ideal	6943 (92.90)	4215 (90.33)	< 0.001	2298 (92.77)	646 (89.23)	0.001	492 (92.66)	469 (88.32)	0.032
Borderline High	444 (5.94)	362 (7.76)		153 (6.18)	59 (8.15)		32 (6.03)	45 (8.47)	
High	87 (1.16)	89 (1.91)		26 (1.05)	19 (2.62)		7 (1.32)	17 (3.20)	
HDL-C level (mmol/L)									
Mean (SD)	1.33 (0.36)	1.38 (0.38)	< 0.001	1.25 (0.32)	1.50 (0.35)	< 0.001	1.24 (0.26)	1.52 (0.35)	< 0.001
HDL-C categories									
Normal	6694 (89.56)	4266 (91.43)	0.001	2149 (86.76)	697 (96.27)	< 0.001	465 (87.57)	511 (96.23)	< 0.001
Low	780 (10.44)	400 (8.57)		328 (13.24)	27 (3.73)		66 (12.43)	20 (3.77)	

^a^ Adjustment Approach enrolled 12,140 individuals met eligibility criteria

^b^ Restriction Approach enrolled 3201 individuals aged 50–53 years

^c^ Matching Approach enrolled 531 age- and gender-matched pairs (1062 individuals)

^d^ The Chi-square test and Student’s t test were used to compare demographic characteristics and behavioral factors in the undernutrition exposed and non-exposed cohorts

To evaluate the effect of early-life undernutrition on long-term risk of dyslipidemia, we compared the prevalent risk of dyslipidemia in the exposed cohort to that of the non-exposed cohort. As shown in Fig. 2A, undernutrition increased the risk of having BHA TC (OR_Adjustment_ = 1.61, 95% CI: 1.37–1.89; OR_Restriction_ = 1.56, 95% CI: 1.27–1.93; OR_Matching_ = 1.87, 95% CI: 1.42–2.47), TG (OR_Adjustment_ = 1.33, 95% CI: 1.13–1.55; OR_Restriction_ = 1.30, 95% CI: 1.05–1.60; OR_Matching_ = 1.34, 95% CI: 1.02–1.76), LDL-C (OR_Adjustment_ = 1.75, 95% CI: 1.35–2.27; OR_Restriction_ = 1.53, 95% CI: 1.10–2.12; OR_Matching_ = 1.77, 95% CI: 1.15–2.73), and dyslipidemia (OR_Adjustment_ = 1.52, 95% CI: 1.31–1.76; OR_Restriction_ = 1.45, 95% CI: 1.19–1.77; OR_Matching_ = 1.60, 95% CI: 1.24–2.06), and these associations were statistically significant over all three approaches. When the outcome was set as high levels of TC, TG, LDL-C, and dyslipidemia (Fig. 2B), similar patterns of association were observed. Regarding HDL-C, an inverse association in undernutrition and risk of low HDL-C was identified (OR_Adjustment_ = 0.34, 95% CI: 0.26–0.43; OR_Restriction_ = 0.27, 95% CI: 0.18–0.42; OR_Matching_ = 0.30, 95% CI: 0.18–0.51, Supplementary Fig. 1). The identified association of undernutrition exposure and risk of dyslipidemia remained in subgroup analysis stratified by BMI groups, especially in participants with BMI ≤ 24 kg/m^2^ (Supplementary Fig. 2).

**Fig. 3:**
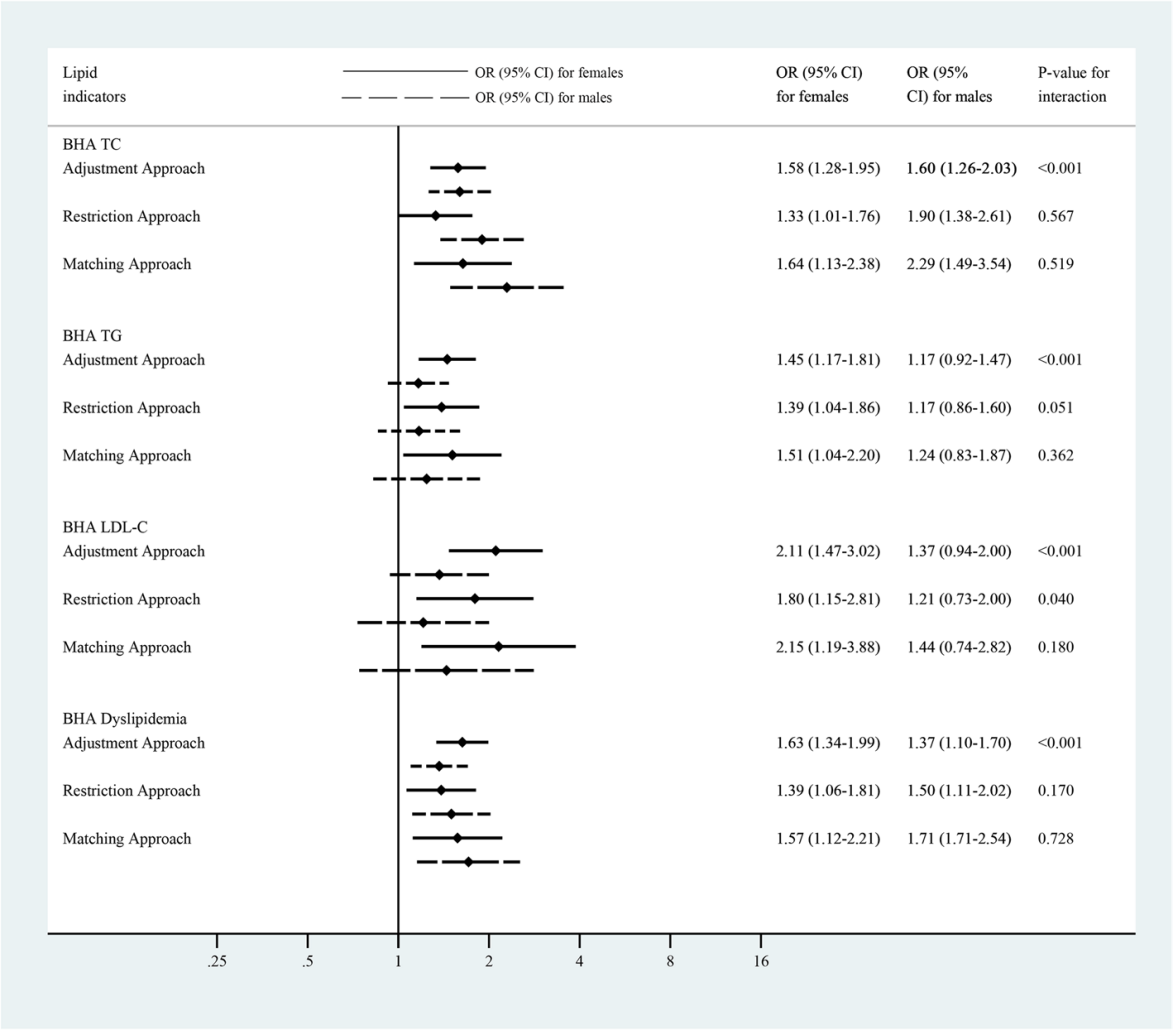
Association of undernutrition and risk of borderline high and above (BHA) dyslipidemia status stratified by gender*. *Interaction between early-life undernutrition and gender was tested by adding the interaction term of the undernutrition variable and gender into the model. ORs were adjusted for age, occupation, BMI, blood pressure, cigarette smoking, alcohol drinking, fried food intake, salty food intake, spicy food intake, heartburn and regurgitation, and self-reported history of diabetes

In the subgroup analysis stratified by gender (Fig. 3, Supplementary Fig. 1, Supplementary Fig. 3), undernutrition was associated with an increased risk of BHA TG and BHA LDL-C in females (TG: OR_Adjustment, female_ = 1.45, 95% CI: 1.17–1.81; OR_Restriction, female_ = 1.39, 95% CI: 1.04–1.86; OR_Matching, female_ = 1.51, 95% CI: 1.04–2.20; LDL-C: OR_Adjustment, female_ = 2.11, 95% CI: 1.47–3.02; OR_Restriction, female_ = 1.80, 95% CI: 1.15–2.81; OR_Matching, female_ = 2.15, 95% CI: 1.19–3.88). However, this association was not identified among males. The gender specific association patterns of undernutrition and TC, HDL-C, and dyslipidemia were not consistent across the three analytic approaches (Fig. 3, Supplementary Fig. 1).

## Discussion

Nutrition is vital to the developmental processes of humans, and undernutrition in early-stage of development may have significant long-term health effects [6–8]. In the current study, we took the Great Chinese Famine as a model for the investigation of the association of severe under-nutrition and risk of dyslipidemia during adulthood. After carefully controlling the confounding effect of age, results of this study confirmed that exposure to undernutrition in early life (being exposed to the Great Famine under the age of 3) increases the prevalent risk of dyslipidemia in adulthood.

A strength of this study lies in the design and analytic approaches. In previous studies, there were mainly two methods used to control the confounding effect of the age variable, namely adjustment and combination [12, 16–19]. The principle of adjustment is to report the age-adjusted prevalence of dyslipidemia by using a multivariable model. However, adjustment was usually not been strong enough, given the fact that there was an “age gap” in the conventional birth cohorts under a cross-sectional design. As such, residual confounding still had the potential to bias the magnitude or even the direction of the observed association. The second method employed an age-appropriate control by combining post- and pre-famine cohorts together. This method may result in exposure contamination that pre-famine cohort individuals (who have been exposed to famine) were assigned into the control group and led to potentially biased estimation. In this study, the baseline investigation was of 5 years duration for the *ESECC* trial, creating a unique situation where the age range was lengthened (50–60 years in the exposed cohort and 43–53 years in the non-exposed cohort) and overlapped at 50–53 years in the two birth cohorts. This age overlap helps strengthen the power of age adjustment in multivariable models under the Adjustment Approach. Moreover, we made use of two other analytical approaches (Restriction and Matching) to reduce the age difference and to make the exposed and non-exposed cohorts more comparable based on this unique “overlapped age range” in the *ESECC* cohort members. Due to these measurements, for the first time we can efficiently reduce the potential confounding effects brought by age.

In this study, we found that early-life undernutrition increased the risk of having BHA and high levels of TC, TG, LDL-C, and dyslipidemia during adulthood, and this confirmed results in several previous studies in China [10, 16, 17, 20]. Hypotheses have been proposed to explain this phenomenon including the thrifty phenotype hypothesis [21–23] and epigenetic dysregulation theory [24, 25]: 1) The “thrifty phenotype” hypothesis proposes that fetal and infant undernutrition would set in train mechanisms of nutritional thrift, which has a differential impact on the growth of different organs, with selective protection of brain growth. The adaptive responses to the nutritional thrift permanently change the structure and function of the body which could be beneficial for early survival (e.g. catch-up growth) but may increase the risk of metabolic disorders especially during a period of adequate or plentiful nutrition in growth. 2) The epigenetic dysregulation theory proposes that epigenetic change is one of the molecular mechanisms behind these epidemiological associations of early-life undernutrition and the risk of metabolic disorders. Exposure to undernutrition in sensitive stages of development may lead to epigenetic changes, which in turn may generate the unique birth phenotype and result in dysregulation in physiological functions.

When stratified by gender, female participants with severe undernutrition experience had an increased risk of BHA TG and LDL-C, but this association was not identified in males. Potential explanations for this gender specific association pattern in undernutrition and BHA TG and LDL-C included: 1) males usually have higher mortality rates under the influence of nutritional deficiency, and as such male survivors tend to be stronger and healthier and less vulnerable to health problems during adulthood [26]. 2) Chinese traditional culture has shown that male offspring may get preferential attention and suffer less severe food shortages as compared with female offspring [27]. For TC, although the *P* value for interaction was < 0.05 in the Adjustment Approach, the absolute difference in estimated ORs between females and males was small (1.58 vs. 1.60) with little biological meaning; and neither of the *P* values of interaction was significant in Restriction and Matching Approaches. Thus, gender difference regarding early-life undernutrition and BHA TC needs to be further investigated.

Heterogeneity of the association pattern was detected in HDL-C and the other three lipid indicators. That is, undernutrition exposure in early life decreased the risk of having reduced HDL-C but increased the risk of high TC, TG, and LDL-C during adulthood. This association of undernutrition and HDL-C has rarely been reported in previous studies. To date, only one study suggested that women with famine experience in adolescence had a significantly decreased prevalence of low HDL-C as compared with non-exposed controls [28]. Previously, we reported prevalence and trends with age were notably different between HDL-C and three other lipid indicators in the *ESECC* population [4]. This revealed HDL-C may have a unique biologic role in the process of lipid metabolism.

Findings from this study have practical implications. First, middle-aged individuals who have been exposed to severe undernutrition in early life are at increased risk of having dyslipidemia, and priority should be given to these individuals in dyslipidemia screening projects. Second, more attention should be paid to the nutritional conditions of vulnerable individuals (e.g. infants and young children) in poverty areas to ensure better population-level primary prevention and management of CVD in their future adulthood.

The study also has limitations. First, this is a single-center study in Henan Province. Although this is an agricultural-dominant area that suffered severe food shortage in 1959–61, results of the current study still require further verification in other populations. Second, the non-exposed cohort defined in this study may not be ideally clean even with a one-year washout period, as recovery from famine is usually slow-paced. Third, unmeasured confounding might still exist although several lifestyle factors were collected and accounted for in this study.

## Conclusions

In summary, we carefully evaluated the association of undernutrition in early life and the risk of dyslipidemia, based on the Great Chinese Famine of 1959–61. Rigorous study design and analytic approaches were applied to control the confounding effect of age. Results from this study demonstrated that undernutrition exposure in early life increased the risk of having high levels of TC, TG, LDL-C, and dyslipidemia, and decreased the risk of having reduced HDL-C in adulthood. The effect of undernutrition on TG and LDL-C abnormalities was evident among females but not observed in males. These results emphasize the importance of nutrition conditionals in early-stage of life to the long-term health consequences.

## Supplementary Information

**Additional file 1: Supplement Table 1**. Definitions and coding forms of risk factors of dyslipidemia investigated in *ESECC* trial from rural Hua County, China. **Supplement Table 2**. Selected demographic and behavioral characteristics in three analytical approaches. **Supplementary Figure 1**. Estimates of association of undernutrition and risk of low HDL-C in pooled analysis and subgroup analysis*. **Supplementary Figure 2**. Association of undernutrition and dyslipidemia stratified by BMI groups in three analytic approaches*. **Supplementary Figure 3**. Association of undernutrition and high status of dyslipidemia stratified by gender in three analytic approaches*.

## Data Availability

The datasets used and/or analysed during the current study are available from the corresponding author on reasonable request.

## Abbreviations

BHA: Borderline high and above
BMI: Body mass index
CVD: Cardiovascular disease
HDL-C: High-density lipoprotein cholesterol
LDL-C: Low-density lipoprotein cholesterol
TC: Total cholesterol
TG: Triglycerides
