# Supplementary material for: Association of early-life undernutrition and risk of dyslipidemia in adulthood: a population-based cohort study
Source: BMC Public Health. 2021 Nov 20;21:2129. doi: 10.1186/s12889-021-12211-8 (PMC8605529; doi:10.1186/s12889-021-12211-8)
Supplement: Supplementary file 1 — Additional file 1: Supplement Table 1. Definitions and coding forms of risk factors of dyslipidemia investigated in ESECC trial from rural Hua County, China. Supplement Table 2. Selected demographic and behavioral characteristics in three analytical approaches. Supplementary Figure 1. Estimates of association of undernutrition and risk of low HDL-C in pooled analysis and subgroup analysis*. Supplementary Figure 2. Association of undernutrition and dyslipidemia stratified by BMI groups in three analytic approaches*. Supplementary Figure 3. Association of undernutrition and high status of dyslipidemia stratified by gender in three analytic approaches*. [file 12889_2021_12211_MOESM1_ESM.docx]

**Supplementary Material**

| Supplement Table 1. Definitions and coding forms of risk factors of dyslipidemia investigated in *ESECC* trial from rural Hua County, China | |
| --- | --- |
| Variable | Definition |
| Gender | Participants were asked of gender. Male was coded as 0 and female coded as 1. |
| Age | Age at enrollment was calculated as Round ((Interview Date-Birthday Date)/365.25). Age groups were categorized by 5-year interval. Subjects aged 45-49 were coded as 0, subjects aged 50-54 coded as 1, subjects aged 55-59 coded as 2, subjects aged 60-64 coded as 3 and subjects aged 65-69 were coded as 4. |
| Occupation | Participants were asked about their occupation in questionnaire. This question had five options: 1) manager; 2) office staff; 3) technician; 4) manual worker; 5) farmer or others. Subjects selecting 1), 2) or 3) were defined as nonphysical worker and coded as 0; and subjects selecting 4) or 5) were defined as physical worker and coded as 1. |
| Body mass index | BMI was calculated as body weight in kilograms divided by the square of body height in meters (kg/m^2^). Subjects were categorized into three groups as BMI ≤ 24 kg/m^2^ (normal, coded as 0), 24 kg/m^2^<BMI<28 kg/m^2^ (overweight, coded as 1) BMI ≥28 kg/m^2^ (obesity, coded as 2). |
| Blood pressure | Blood pressure was measured for each participant. Subjects with systolic blood pressure ≥ 140 mmHg or diastolic blood pressure ≥ 90 mmHg were defined as hypertensive and coded as 1; others were defined as non-hypertensive and coded as 0. |
| Smoking | Participants were asked whether they smoked and detailed period and quantity of smoking in questionnaire. Total amount of cigarette consumption was calculated as period multiply quantity of smoking. Smoking group was categorized by quartiles of accumulative consumption. Subjects who didn't smoke were coded as 0; subjects with consumption in Q1-Q3 were defined as moderate-smoker and coded as 1; subjects with consumption in Q4 was defined as heavy-smoker and coded as 2. |
| Alcohol drinking | Participants were asked whether they drunk and detailed period and quantity in questionnaire. Total amount of alcohol consumption was calculated as period multiply quantity of drinking. Alcohol drinking group was categorized by quartiles of accumulative consumption. Subjects who didn't drink were coded as 0; subjects with consumption in Q1-Q3 were defined as moderate-drinker and coded as 1; subjects with consumption in Q4 was defined as heavy-drinker and coded as 2. |
| Fried food intake | Participants were asked about the frequency of fried food intake in questionnaire. This question had three options: 1) seldom (<1 time a week); 2) occasionally (1-3 times a week); 3) often (≥4 times a week). Subjects selecting 1) were coded as 0 and subjects selecting 2) or 3) were coded as 1. |
| Salty food intake | Participants were asked about the frequency of salty food intake in questionnaire. This question had three options: 1) seldom (<1 time a week); 2) occasionally (1-3 times a week); 3) often (≥4 times a week). Subjects selecting 1) were coded as 0 and subjects selecting 2) or 3) were coded as 1. |
| Heartburn and regurgitation | Participants were asked whether they had the symptom of heartburn and regurgitation in questionnaire. This question had two options: 1) no; 2) yes. Subjects selecting 1) were coded as 0 and subjects selecting 2) were coded as 1. |
| Self-reported history of diabetes | Participants were asked about their medical history in questionnaire. Subjects reporting diabetes history were coded as 1 and others were coded as 0. |

| Supplement Table 2. Selected demographic and behavioral characteristics in three analytical approaches | | | | | | | | | | | |
| --- | --- | --- | --- | --- | --- | --- | --- | --- | --- | --- | --- |
|  | Adjustment Approach^a^ | | |  | Restriction Approach^b^ | | |  | Matching Approach^c^ | | |
|  | Non-exposed cohort (N=7474) | Exposed cohort (N=4666) | *p* value^d^ |  | Non-exposed cohort (N=2477) | Exposed cohort (N=724) | *p* value^d^ |  | Non-exposed cohort (N=531) | Exposed cohort (N=531) | *p* value^d^ |
| Body mass index |  |  |  |  |  |  |  |  |  |  |  |
| ≤24 kg/m^2^ | 2376 (31.79) | 1839 (39.41) | <0.001 |  | 746 (30.12) | 320 (44.20) | <0.001 |  | 170 (32.02) | 234 (44.07) | <0.001 |
| 24.1-27.9 kg/m^2^ | 3214 (43.00) | 1897 (40.66) |  |  | 1101 (44.45) | 265 (36.60) |  |  | 223 (42.00) | 199 (37.48) |  |
| ≥28 kg/m^2^ | 1884 (25.21) | 930 (19.93) |  |  | 630 (25.43) | 139 (19.20) |  |  | 138 (25.99) | 98 (18.46) |  |
| Blood pressure |  |  |  |  |  |  |  |  |  |  |  |
| Nonhypertension | 3803 (50.88) | 2084 (44.66) | <0.001 |  | 1090 (44.00) | 407 (56.22) | <0.001 |  | 214 (40.30) | 299 (56.31) | <0.001 |
| Hypertension | 3671 (49.12) | 2582 (55.34) |  |  | 1387 (56.00) | 317 (43.78) |  |  | 317 (59.70) | 232 (43.69) |  |
| Spicy food intake |  |  |  |  |  |  |  |  |  |  |  |
| Seldom | 4511 (60.36) | 2970 (63.65) | <0.001 |  | 1604 (64.76) | 417 (57.60) | <0.001 |  | 338 (63.65) | 304 (57.25) | 0.033 |
| Often | 2963 (39.64) | 1696 (36.35) |  |  | 873 (35.24) | 307 (42.40) |  |  | 193 (36.35) | 227 (42.75) |  |
| Occupation |  |  |  |  |  |  |  |  |  |  |  |
| Physical worker | 7269 (97.26) | 4572 (97.99) | 0.012 |  | 2421 (97.74) | 702 (96.96) | 0.232 |  | 520 (97.93) | 514 (96.80) | 0.250 |
| Nonphysical worker | 205 (2.74) | 94 (2.01) |  |  | 56 (2.26) | 22 (3.04) |  |  | 11 (2.07) | 17 (3.20) |  |
| Smoking |  |  |  |  |  |  |  |  |  |  |  |
| No | 5162 (69.07) | 3068 (65.75) | <0.001 |  | 1663 (67.14) | 497 (68.65) | 0.521 |  | 354 (66.67) | 355 (66.85) | 0.770 |
| Moderate amount | 1949 (26.08) | 1255 (26.90) |  |  | 666 (26.89) | 180 (24.86) |  |  | 148 (27.87) | 142 (26.74) |  |
| Large amount | 363 (4.86) | 343 (7.35) |  |  | 148 (5.97) | 47 (6.49) |  |  | 29 (5.46) | 34 (6.40) |  |
| Alcohol drinking |  |  |  |  |  |  |  |  |  |  |  |
| No | 5795 (77.54) | 3631 (77.82) | 0.591 |  | 1929 (77.88) | 568 (78.45) | 0.560 |  | 411 (77.40) | 410 (77.21) | 0.992 |
| Moderate amount | 1294 (17.31) | 781 (16.74) |  |  | 419 (16.92) | 113 (15.61) |  |  | 88 (16.57) | 88 (16.57) |  |
| Large amount | 385 (5.15) | 254 (5.44) |  |  | 129 (5.21) | 43 (5.94) |  |  | 32 (6.03) | 33 (6.21) |  |
| Fried food intake |  |  |  |  |  |  |  |  |  |  |  |
| Seldom | 5215 (69.78) | 3351 (71.82) | 0.016 |  | 1730 (69.84) | 503 (69.48) | 0.850 |  | 364 (68.55) | 372 (70.06) | 0.595 |
| Often | 2259 (30.22) | 1315 (28.18) |  |  | 747 (30.16) | 221 (30.52) |  |  | 167 (31.45) | 159 (29.94) |  |
| Salty food intake |  |  |  |  |  |  |  |  |  |  |  |
| Seldom | 1595 (21.34) | 975 (20.90) | 0.559 |  | 568 (22.93) | 160 (22.10) | 0.639 |  | 140 (26.37) | 118 (22.22) | 0.115 |
| Often | 5879 (78.66) | 3691 (79.10) |  |  | 1909 (77.07) | 564 (77.90) |  |  | 391 (73.63) | 413 (77.78) |  |
| Heartburn and regurgitation | |  |  |  |  |  |  |  |  |  |  |
| No | 5460 (73.05) | 3401 (72.89) | 0.843 |  | 1852 (74.77) | 523 (72.24) | 0.171 |  | 406 (76.46) | 377 (71.00) | 0.043 |
| Yes | 2014 (26.95) | 1265 (27.11) |  |  | 625 (25.23) | 201 (27.76) |  |  | 125 (23.54) | 154 (29.00) |  |
| Self-reported diabetes history | |  |  |  |  |  |  |  |  |  |  |
| No | 7367 (98.57) | 4564 (97.81) | 0.002 |  | 2429 (98.06) | 713 (98.48) | 0.461 |  | 521 (98.12) | 522 (98.31) | 0.817 |
| Yes | 107 (1.43) | 102 (2.19) |  |  | 48 (1.94) | 11 (1.52) |  |  | 10 (1.88) | 9 (1.69) |  |
|  |  |  |  |  |  |  |  |  |  |  |  |
| ^a^ Adjustment Approach enrolled 12,140 individuals meeting the eligibility criteria. | | | | | | |  |  |  |  |  |
| ^b^ Restriction Approach enrolled 3,201 individuals aged 50-53 years. | | | | | |  |  |  |  |  |  |
| ^c^ Matching Approach enrolled 531 age- and gender-matched pairs (1,062 individuals). | | | | | | |  |  |  |  |  |
| ^d^ The Chi-square test and Student's t test were used to compare demographic characteristics and behavioral factors between the undernutrition exposed and non-exposed cohorts. | | | | | | | | | | | |


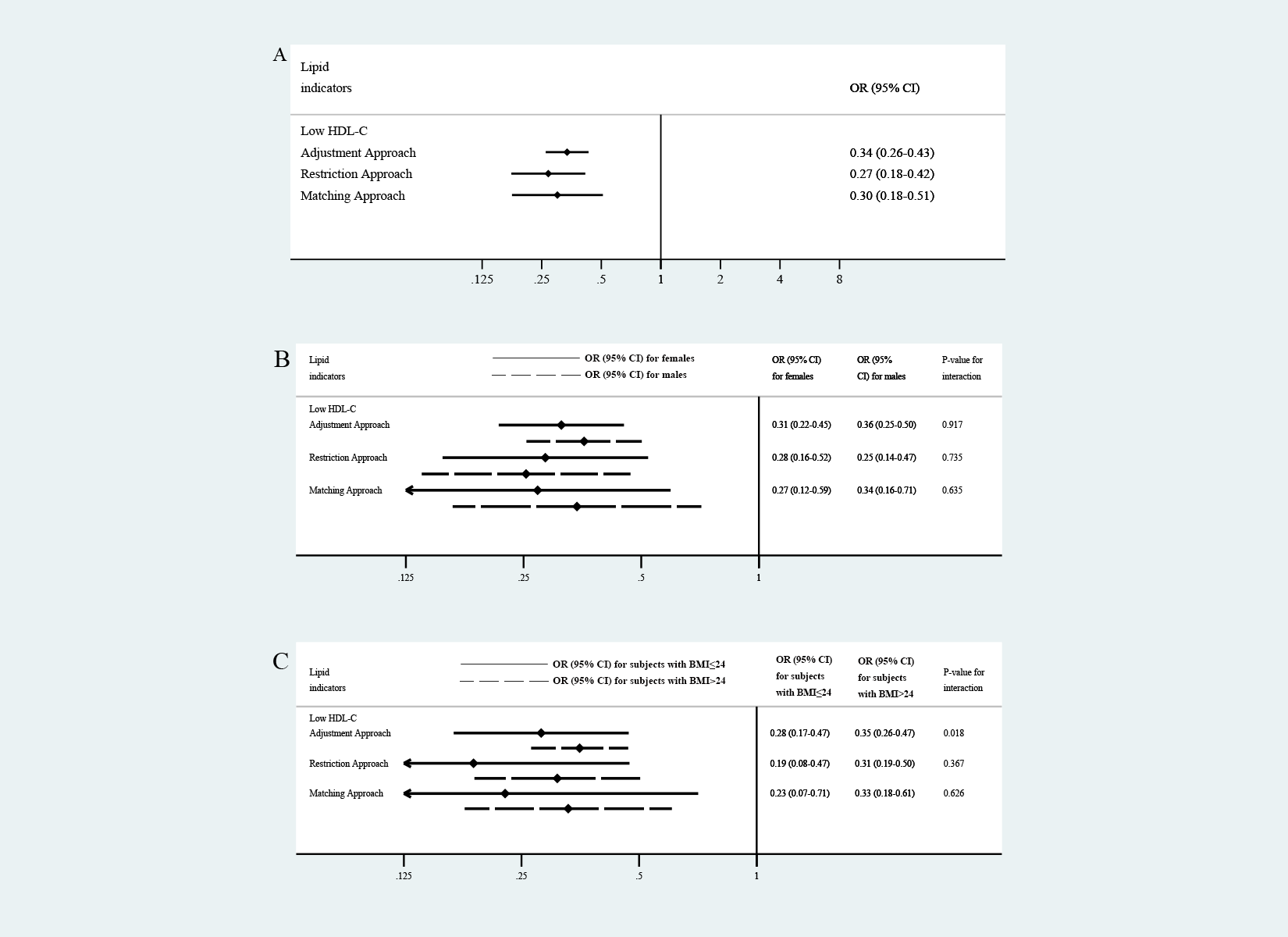


Supplementary Figure 1. Estimates of association of undernutrition and risk of low HDL-C in pooled analysis and subgroup analysis*

A. The effect estimates of undernutrition and risk of low HDL-C in pooled analysis

B. The effect estimates of undernutrition and risk of low HDL-C in subgroup analysis stratified by gender

C. The effect estimates of undernutrition and risk of low HDL-C in subgroup analysis stratified by BMI groups

*ORs were adjusted for age, occupation, BMI, blood pressure, cigarette smoking, alcohol drinking, fried food intake, salty food intake, spicy food intake, heartburn and regurgitation, and self-reported history of diabetes


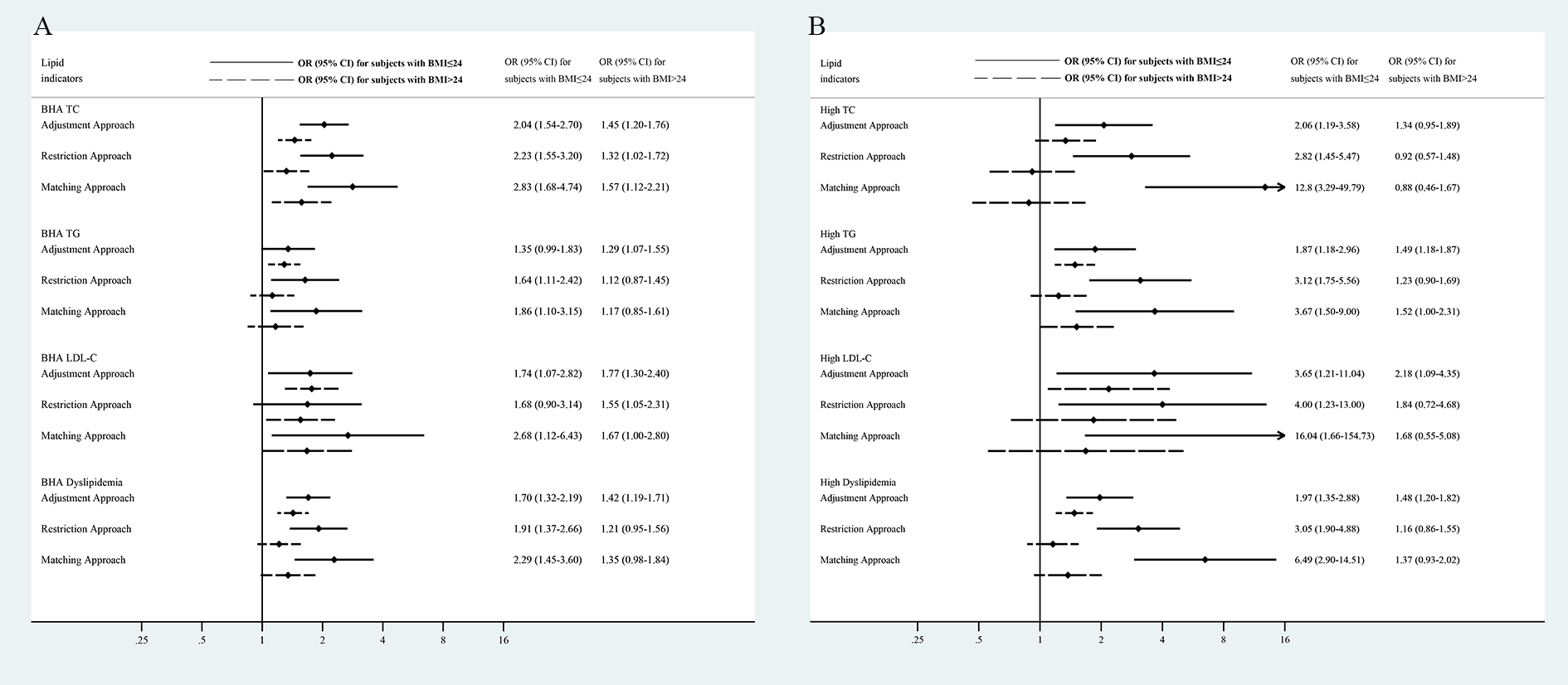


Supplementary Figure 2. Association of undernutrition and dyslipidemia stratified by BMI groups in three analytic approaches*

A. The association of undernutrition and borderline high and above (BHA) dyslipidemia in subgroup analysis stratified by BMI groups

B. The association of undernutrition and high dyslipidemia in subgroup analysis stratified by BMI groups

*ORs were adjusted for age, occupation, BMI, blood pressure, cigarette smoking, alcohol drinking, fried food intake, salty food intake, spicy food intake, heartburn and regurgitation, and self-reported history of diabetes


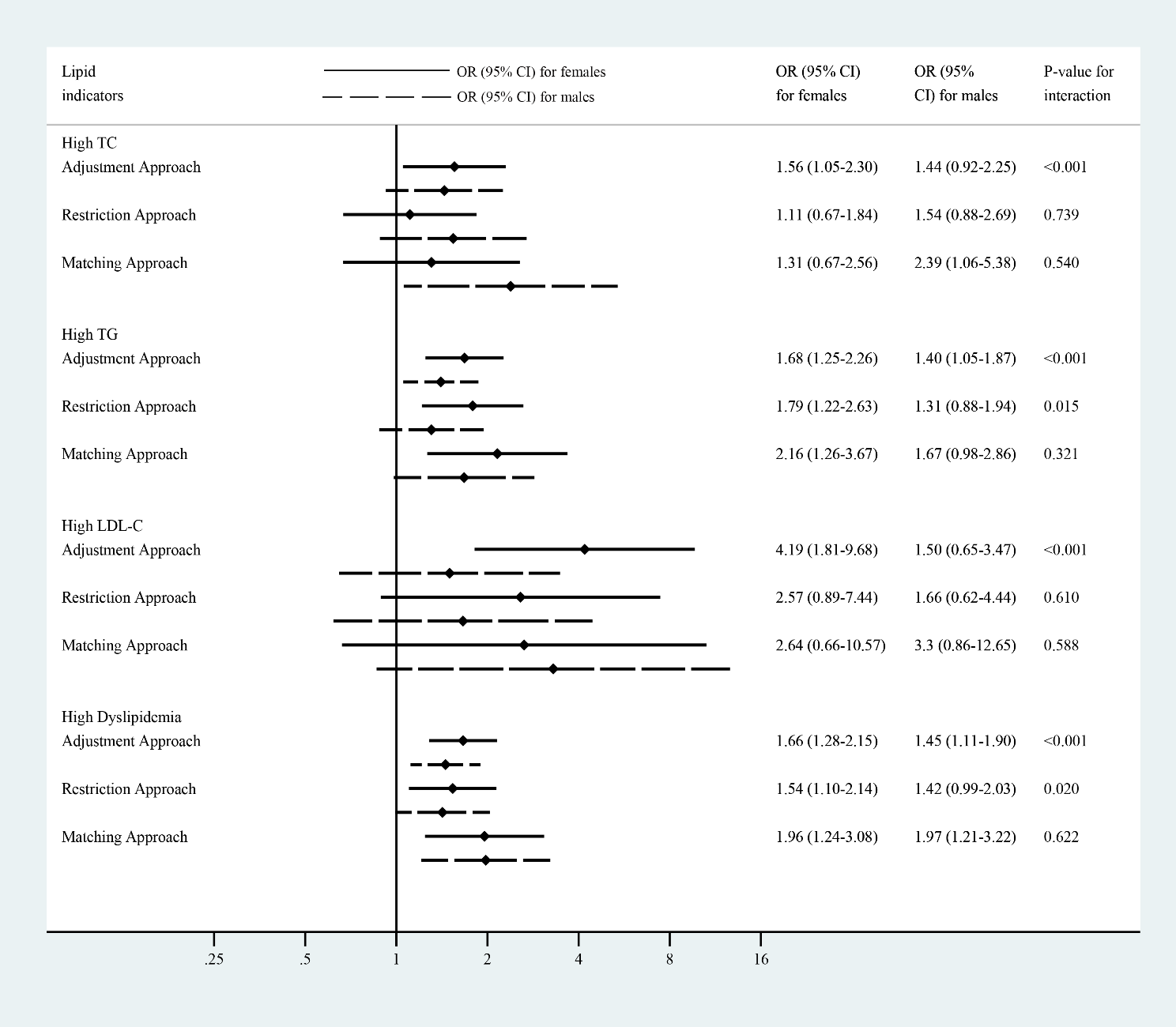


Supplementary Figure 3. Association of undernutrition and high status of dyslipidemia stratified by gender in three analytic approaches*

*ORs were adjusted for age, occupation, BMI, blood pressure, cigarette smoking, alcohol drinking, fried food intake, salty food intake, spicy food intake, heartburn and regurgitation, and self-reported history of diabetes. Interaction between early-life undernutrition exposure and gender was tested by adding the interaction term of the undernutrition variable and gender into the model
